# EVALUATING AGING IN PLACE LONG-TERM CARE REFORMS: A SCOPING REVIEW

**DOI:** 10.1093/geroni/igad104.3308

**Published:** 2023-12-21

**Authors:** Janet MacNeil Vroomen, Isabelle Vullings, Miranda Langendam, Joost Wammes, Dionne Kringos, Bram Wouterse, Emiel Hoogendijk

**Affiliations:** Amsterdam University Medical Centers, Amsterdam, Noord-Holland, Netherlands; Amsterdam University Medical Centers, Amsterdam, Noord-Holland, Netherlands; Amsterdam University Medical Centers, Amsterdam, Noord-Holland, Netherlands; Amsterdam University Medical Centers, Amsterdam, Noord-Holland, Netherlands; Amsterdam University Medical Centers, Amsterdam, Noord-Holland, Netherlands; Erasmus University Rotterdam, Rotterdam, Zuid-Holland, Netherlands; Amsterdam University Medical Centers, Amsterdam, Noord-Holland, Netherlands

## Abstract

There is a global trend of reforming long-term care to enable aging in place making it one of the most pressing health policy issues in both high- and low-middle income countries. However, there is no agreement on what is a successful aging in place reform. A holistic perspective that includes clinical, social and economic indicators is required to evaluate these reforms. In this scoping review, we aim to determine the extent of literature on evaluating aging in place reforms. We used the guidance of the JBI Scoping Review Methodology Group to conduct the scoping review. Articles on evaluation of aging in place reform and reporting on any outcome, process or structure quality indicator were eligible for inclusion. The search strategy was performed in Ovid Medline, Ovid Embase, and Ebscohost Academic Search Premier. There were no restrictions on publication year, language, and study design. The search identified 8,107 records. A total of 84 articles met the inclusion criteria and were included in the scoping review. Most studies were quantitative, observational studies from high income countries, using national population level data or larger cohorts. Frequently reported indicators were: total expenditures on long-term care, program spending per capita, hospitalization rates, length of stay, and home care utilization. Less reported were older adult and informal caregiver related indicators like: health, well-being, informal caregiver hours, and sleeping hours. Policy makers can use our findings to develop appropriate strategies to evaluate and monitor their own aging in place reforms.

